# A Natural Approach to Bell's Palsy: An Osteopathic Treatment Option

**DOI:** 10.7759/cureus.67334

**Published:** 2024-08-20

**Authors:** Nicole Schneider, Susana Shih, Lily Rundquist, Lis Llanio, Asha Kurian, Ashley Ring, Patrick Barry

**Affiliations:** 1 Medicine, Dr. Kiran C. Patel College of Osteopathic Medicine, Nova Southeastern University, Fort Lauderdale, USA; 2 Osteopathic Medicine, Dr. Kiran C. Patel College of Osteopathic Medicine, Nova Southeastern University, Fort Lauderdale, USA

**Keywords:** bell's palsy, bell's palsy treatment, osteopathic manipulative treatment (omt), neurology, osteopathic manipulative medicine (omm)

## Abstract

Bell's palsy (BP) is a rapid-onset neurological disorder causing unilateral facial paralysis, affecting approximately 40,000 people annually in the United States. Suggested treatments for BP include corticosteroids, facial therapy, and osteopathic manipulative treatment (OMT) in order to improve symptoms; however, some people with BP have spontaneous resolution. A 52-year-old female with left-sided facial paralysis and drooping for the past four months due to BP presented to the osteopathic treatment center. For the first three weeks after developing BP, the patient had soreness when attempting to move her facial features, but on later treatments, she only experienced weakness on the left side of her face. The patient's facial sensation was intact bilaterally, but she was unable to move her left eyebrow, eyelid, cheek, and lip. OMT focused on the intraoral musculature, the cervical spine, and cranial treatment utilizing osteopathic techniques such as osteopathic cranial manipulative medicine (OCMM), direct myofascial release, soft tissue, balanced ligamentous tension, and muscle energy. Utilizing the Facial Disability Index (FDI) questionnaire and the Sunnybrook facial grading system (SFGS), an improvement in facial paralysis was seen due to both OMT and physical therapy (PT) treatments. It is difficult to discern which treatments helped the patient the most (OMT, PT, or at-home exercises); however, the patient's improvement was notable. This case study demonstrates that OMT, PT, and at-home exercises may positively contribute toward the improvement of BP symptoms by addressing cranial and muscular somatic dysfunctions of the head and neck. The treatment, which included techniques such as muscle energy and intraoral myofascial release, resulted in significant improvements in facial function and grading scores. One limitation of the study is that, however unlikely, chronic BP may resolve spontaneously, which may have contributed to the patient's progress. While OMT, PT, and at-home exercises contributed to the patient's recovery, further research is needed to substantiate the effectiveness of OMT, PT, and at-home exercises in treating BP.

## Introduction

Bell's palsy (BP) is a rapid, non-progressive neurological disorder affecting an estimated 40,000 people annually in the United States [1]. This condition causes partial or complete unilateral paralysis of the facial nerve (cranial nerve 7), leading to symptoms such as impaired lacrimation and salivation, dysgeusia, and hyperacusis [2]. It is proposed to be triggered idiopathically or by inflammatory processes including trauma, tumors, infection (such as herpes zoster), and conditions such as diabetes mellitus or pregnancy, leading to edema and compression of the facial nerve; however, its etiology is not fully understood, and it is classified as a diagnosis of exclusion [1,3]. In 70% of patients, symptoms spontaneously resolve quickly, within days to weeks. Approximately 30% of patients have delayed recovery and may need additional treatment such as corticosteroids, facial therapy, or botulinum toxin [1,2]. Osteopathic manipulative treatment (OMT) is another potentially viable modality to help relieve irritation and compression of the facial nerve to facilitate function and improve facial weakness [4]. OMT is a treatment modality that addresses muscles, bones, and fascial planes in order to reposition the body to enhance its self-healing properties [5].

A previous case study published in 2020 demonstrated improvement of right-sided BP symptoms in a 32-year-old female through OMT [4]. This study included solely OMT, focusing on lymphatic drainage techniques, osteopathic cranial manipulative medicine (OCMM), counterstain, and muscle energy for a patient with non-improving BP symptoms present for three months [4]. The House-Brackmann scale revealed improvement in her BP symptoms [4]. Larger studies with BP patients treated with pharmacotherapy, PT, OMT, or a combination of these are necessary in determining the most effective treatment approaches.

A systematic review evaluating seven new randomized controlled trials, nine observational studies, and three quasi-experimental or pilot studies found evidence to support the use of facial exercise therapy in patients with facial palsy [6]. However, limitations in this evidence arose due to differences in research design, the interventions evaluated, and the patient populations [6]. The diverse nature of their evidence base restricts the ability to clearly correlate the effectiveness of facial exercise therapy with the time since the onset of facial palsy, clinical severity, or other patient demographics [6]. Despite its limitations, the study provides evidence that facial exercise therapy can improve BP overall. This case study may add further evidence of OMT being one of the treatment modalities that can positively impact chronic BP symptoms.

## Case presentation

A 52-year-old Caucasian female presented to the Osteopathic Treatment Center (OTC) at Nova Southeastern University with a complaint of paralysis and droop of the left side of her face for four months. The patient reported recovering from a common cold prior to the onset of symptoms. Her cold lasted four days, and the facial drooping occurred one week later while brushing her teeth. She noticed the left side of her face had stopped moving and went to the emergency room, where she was sent for imaging. Magnetic resonance imaging (MRI) of the brain showed no abnormalities, and she was sent home with the diagnosis of BP and prescribed prednisone and valacyclovir for 10 days. She went to her primary care physician after finishing her antiviral and was prescribed a second antiviral, acyclovir, along with prednisone for 10 more days. She was then sent to physical therapy (PT) five weeks after her initial symptoms began for a total of 28 weeks, where she performed exercises and used an electrical muscle stimulation (e-stim) machine at a low level. Initially, she went to PT two times a week, which later decreased to once a week, followed by once every two weeks. Four months after symptoms occurred, she presented to the OTC for a total of 17 weeks of overlap between PT and OMT.

She noted pain for the first three weeks of paralysis, followed by soreness when trying to move her facial features or touching her face. In the mornings, her left cheek felt tight. While eating, her left cheek shifted upward, causing her vision to be partially obscured. The patient had a fully intact sensation on her face; however, she experienced tingling and was unable to move her features on the left side. She also noted severe sensitivity to sound for two to three weeks, where she could not tolerate the sound of even a toilet flushing due to pain felt around her left ear. She had no taste and was unable to breathe out of her left nostril. When she coughed or sneezed, she had a decrease in sensitivity internally on the left side. She also had left-eye dryness, requiring the use of over-the-counter eye drops.

The patient had no significant past medical history besides a cesarean section 16 years ago and a rhinoplasty at 24 years old. During physical examination, the cranial nerves 2-12, with the exception of cranial nerves 7 and 12, were intact bilaterally. Weakness was observed in the left eyebrow, eyelid, cheek, and lip, indicating impairment of the facial nerve (cranial nerve 7). Additionally, the tongue deviated to the left, suggesting impairment of the hypoglossal nerve (cranial nerve 12). Muscle strength was 5/5 bilaterally for the upper and lower extremities.

Course of treatment

The patient was seen five times, monthly over the course of five months at the OTC with OMT primarily focused on intraoral musculature, the cranium, and the cervical spine. The techniques employed included OCMM, direct myofascial release, soft tissue, balanced membranous tension (BMT), balanced ligamentous tension (BLT), articulatory, muscle energy, and Ruddy's technique. Ruddy's technique improves the fluctuation of aqueous humor through gentle tapotement of the upper eyelid. The physician's second and third digits are placed on top of the patient's closed eye, and tapotement is performed with the physician's other index finger for 30-60 seconds [7].

Somatic dysfunctions (Table 1) that were diagnosed included a hypertonic left masseter, hypertonic left medial and lateral pterygoids, internally rotated left zygoma, internally rotated left temporal bone, a right sidebending rotation cranial strain pattern, left eyelid fascial restriction, atlanto-occipital (OA) joint compression, extended, sidebent right and rotated left, cervical vertebrae two to four extended, rotated and sidebent left, hypertonic scalenes bilaterally, hypertonic trapezius bilaterally, and hypertonic levator scapulae bilaterally.

**Table 1:** Somatic dysfunctions OA: atlanto-occipital; ESrRl: extended, sidebent right, rotated left; C2-4: cervical vertebrae 2-4; ERlSl: extended, sidebent left, rotated left

Body Region	Somatic Dysfunction
Head	Hypertonic left masseter
	Hypertonic left medial and lateral pterygoids
	Left internally rotated zygoma
	Left internally temporal bone
	Right sidebending rotation
	Left eyelid fascial restriction
	OA joint compression
	OA ESrRl
Neck	C2-4 ERlSl
	Hypertonic scalenes bilaterally
	Hypertonic trapezius bilaterally
	Hypertonic levator scapulae bilaterally

All the OMT sessions involved treatment of the cranium and cervical regions. OA decompression was performed, followed by BLT of the cervical spine. Muscle energy and soft tissue were utilized when treating the hypertonic scalenes, levator scapulae, and trapezius bilaterally. OCMM was implemented by performing venous sinus drainage, temporal decompression, frontal lift, parietal lift, sphenoid lift, fourth ventricle compression (CV4), and BMT on the right sidebending rotation cranial strain pattern. Ruddy's technique was included in two of the visits due to left eyelid restriction. While this technique is primarily used to improve the fluctuation of the aqueous humor, it was used in this patient to also decrease the tension in the eyelid. Finally, intraoral techniques were used to treat the left hypertonic masseter, zygoma, and medial and lateral pterygoids with direct myofascial release and soft tissue.

The patient was advised to perform at-home massages to improve muscle tightness. She was taught to first open the thoracic inlet by massaging the areas superior and inferior to the clavicles. The patient was instructed to externally massage her hypertonic masseters bilaterally by opening her jaw and sidebending her head to the side being treated while slowly moving her knuckles superior to inferior along the muscle three times. It was advised she massage her scalp bilaterally to release fascial tension. The patient reported noticeable improvement in the tightness of her left face after incorporating the daily massage routine.

## Discussion

Prior to each OMT session, with the exception of the first visit in January, the Facial Disability Index (FDI) questionnaire was completed by the patient [8]. The Sunnybrook facial grading system (SFGS) was also utilized at the patient's PT sessions to evaluate facial symmetry at rest and with voluntary movement, along with the presence of synkinesis [9]. These forms showed an improvement in the patient's facial paralysis following OMT and PT.

**Figure 1:**
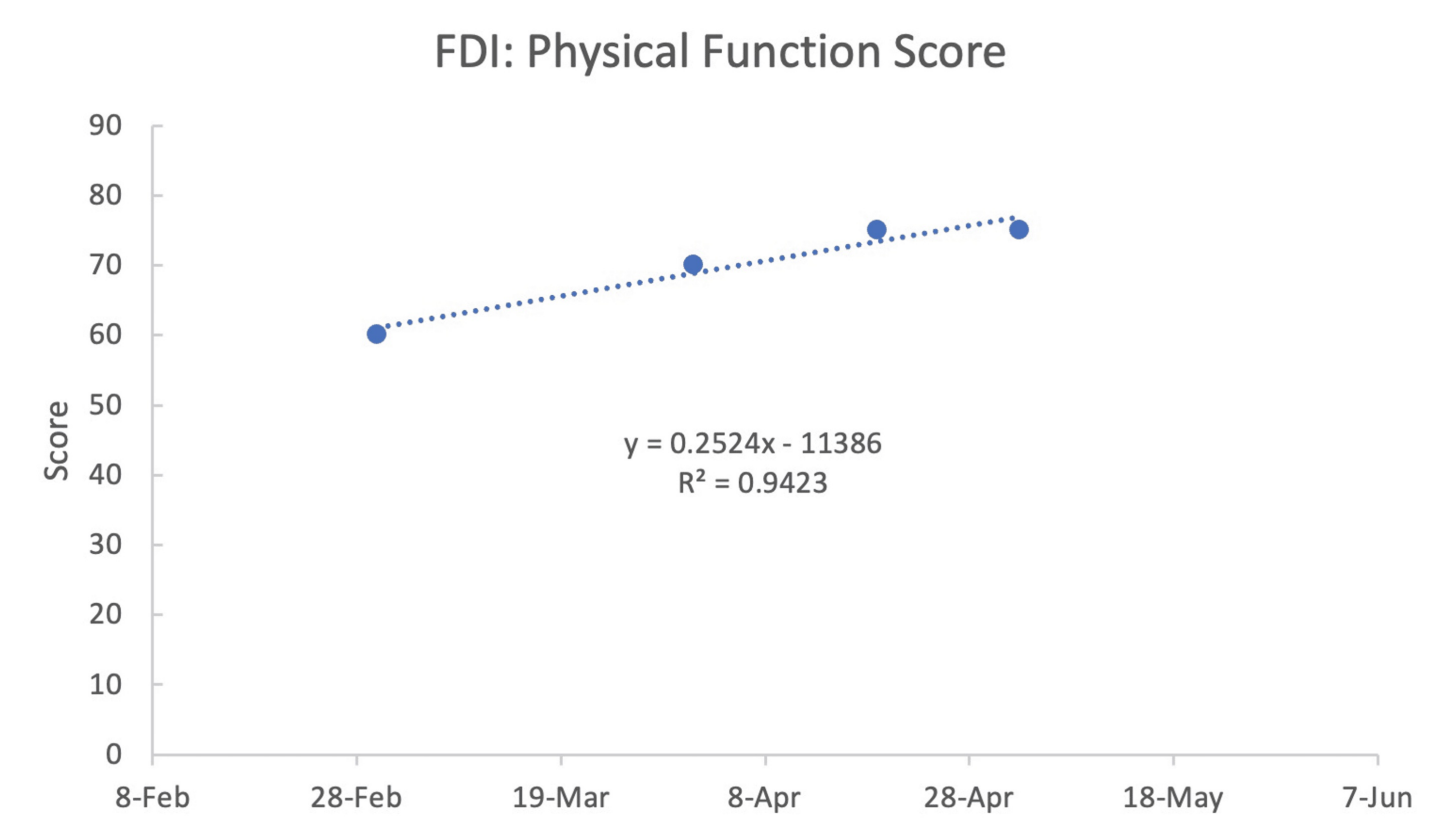
Facial disability index (FDI): physical function score The larger the number, the better the patient's symptoms.

**Figure 2:**
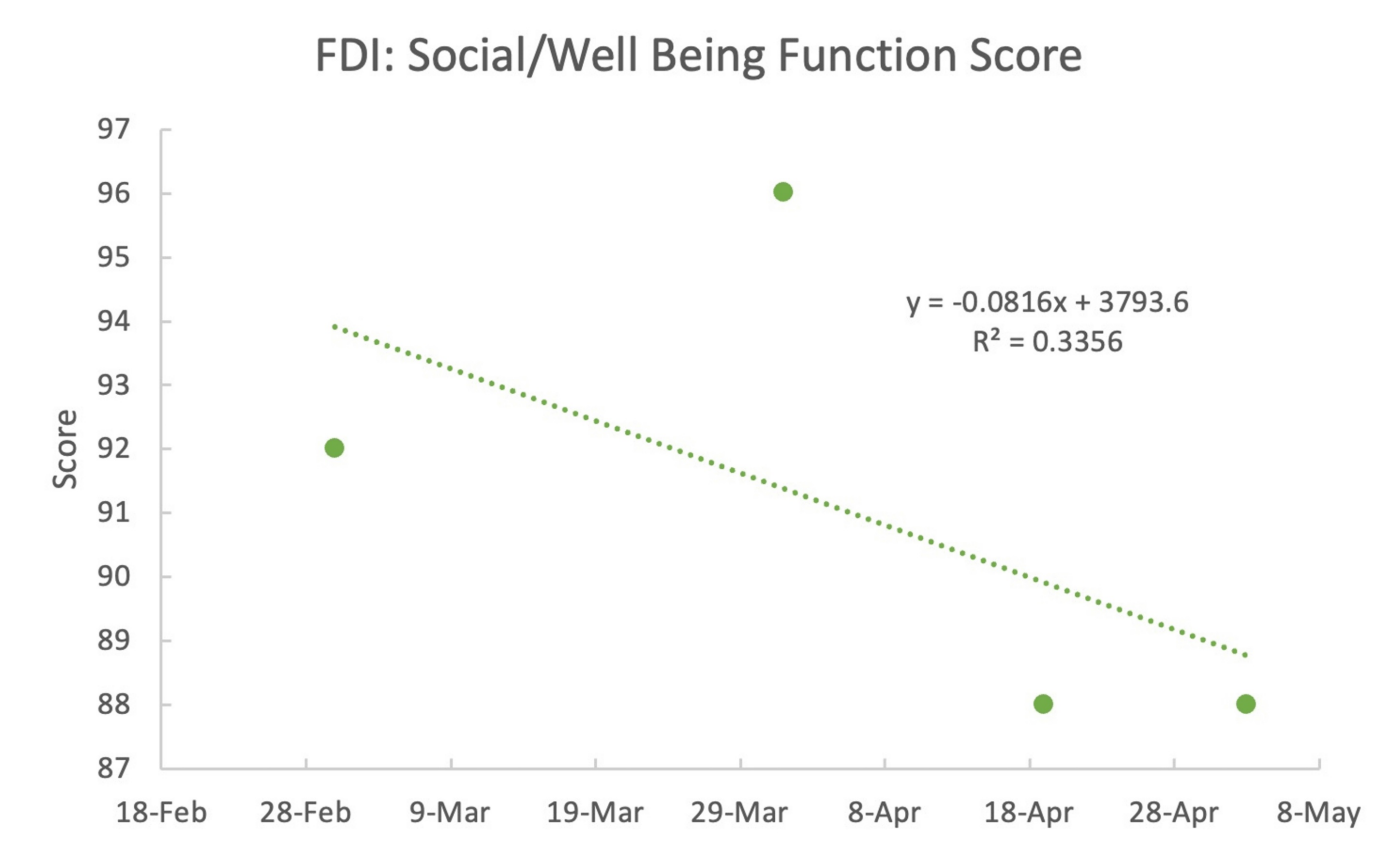
Facial disability index (FDI): social/well-being function score The larger the number, the better the patient's symptoms.

The FDI physical function scores (Figure 1) continued to improve with each visit. The physical function portion of the questionnaire included difficulty keeping food in one's mouth, drinking from a cup, saying specific words, eye tearing or becoming dry, and brushing/rinsing one's teeth. However, the social/well-being function of the FDI had a decrease in improvement (Figure 2). That portion of the questionnaire included the amount of time one felt calm or peaceful, how much time one isolated oneself from people, irritability toward others, how often one wakes up during the night, and if one's facial function had kept them from going out socially. These scores suggested that although the patient was making physical progress, improving socially was challenging due to the prolonged duration of partial facial paralysis as expressed by the patient.

**Figure 3:**
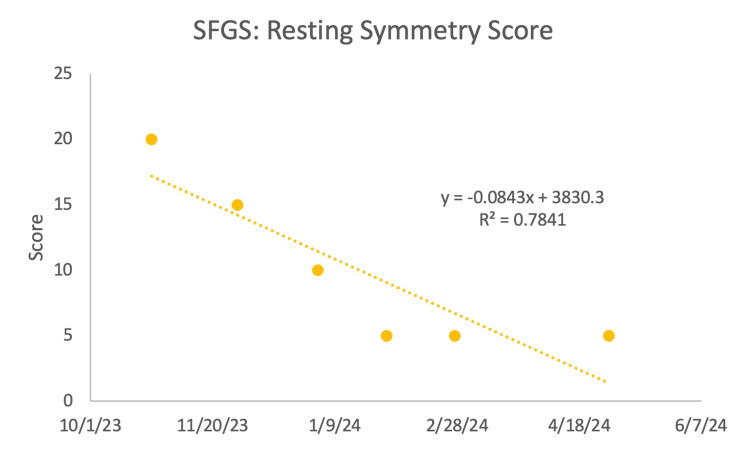
Sunnybrook facial grading system (SFGS): resting symmetry score The smaller the number, the better the patient's symptoms.

**Figure 4:**
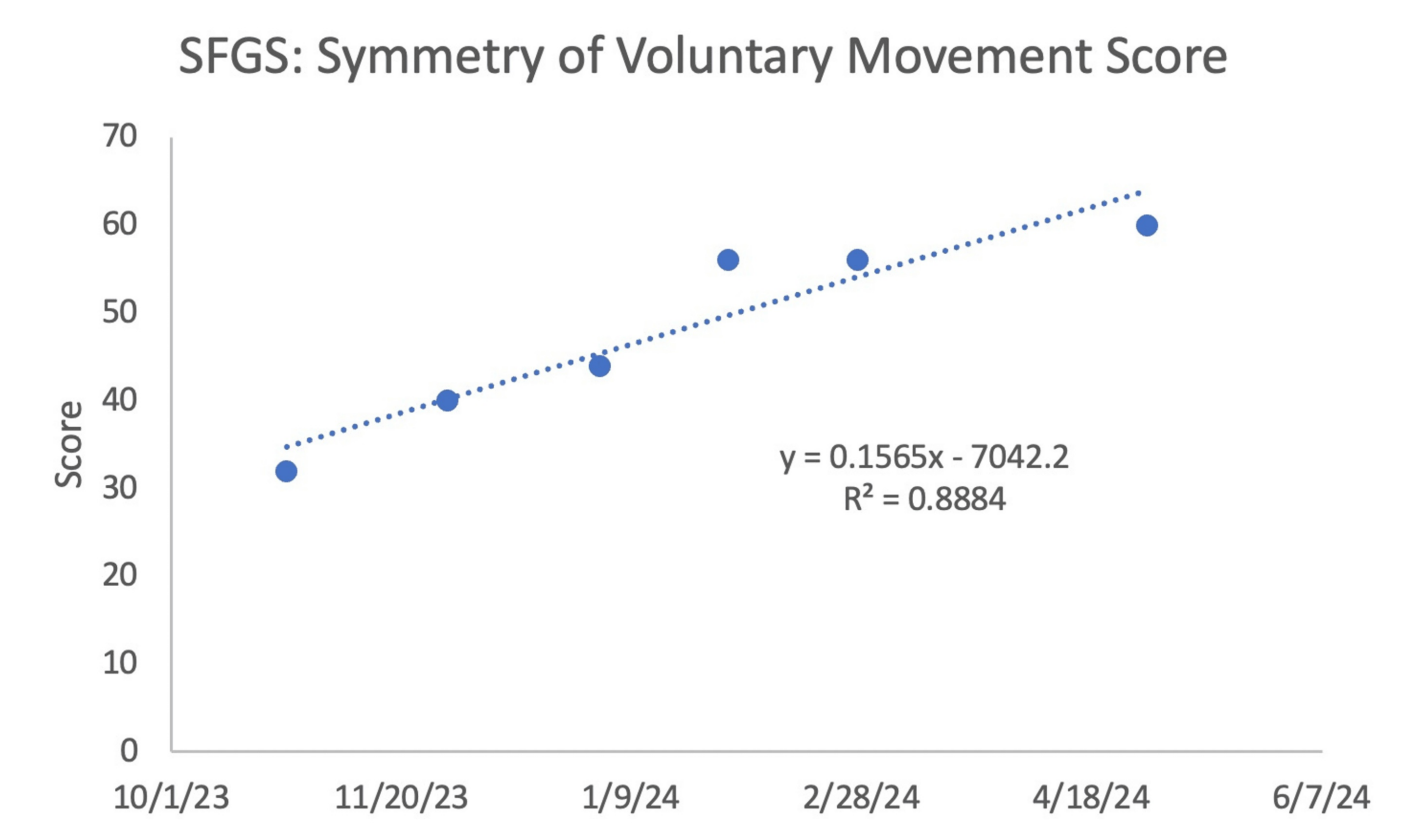
Sunnybrook facial grading system (SFGS): symmetry of voluntary movement score The larger the number, the better the patient's symptoms.

**Figure 5:**
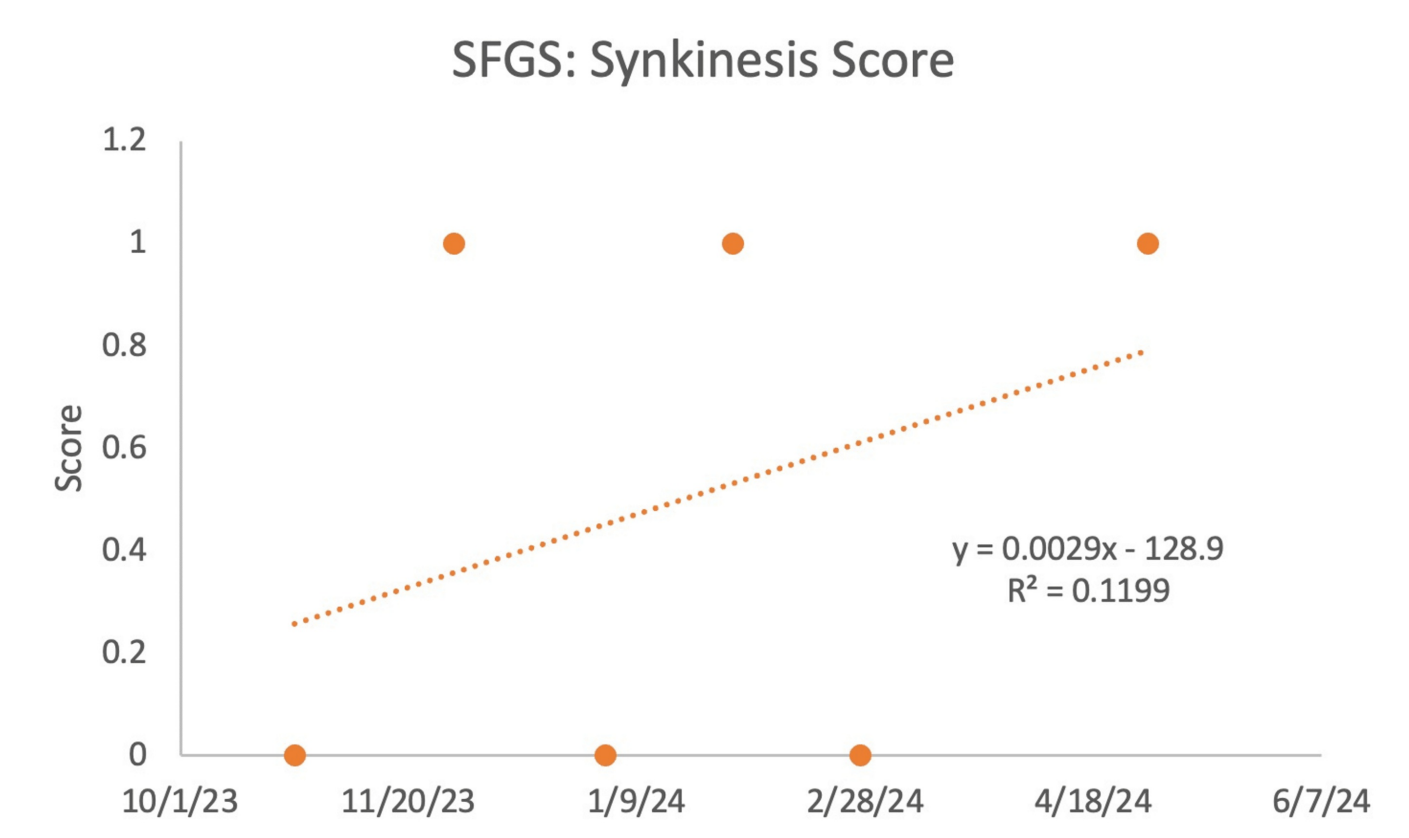
Sunnybrook facial grading system (SFGS): synkinesis score The smaller the number, the better the patient's symptoms.

The SFGS included three components, beginning with resting symmetry, which involved comparison of the eyes, cheeks, and mouth of the paralyzed side to the normal side. An improvement was seen (Figure 3) as the score decreased, indicating increased symmetry in the patient's resting face. The next component was symmetry of voluntary movement, which compared the paralyzed side to the normal side by looking at the degree of muscle excursion: brow lifting, gentle eye closure, open mouth smile, snarl, and lip puckering. There was an increase in values (Figure 4), which showed that the patient's voluntary movement became more symmetrical over the course of treatment. The final component was synkinesis, which measured the degree of involuntary muscle contraction that occurred with each of the different facial expressions. There was no observable change, given that there was fluctuating improvement followed by a decline repeatedly, throughout the treatment (Figure 5).

**Figure 6:**
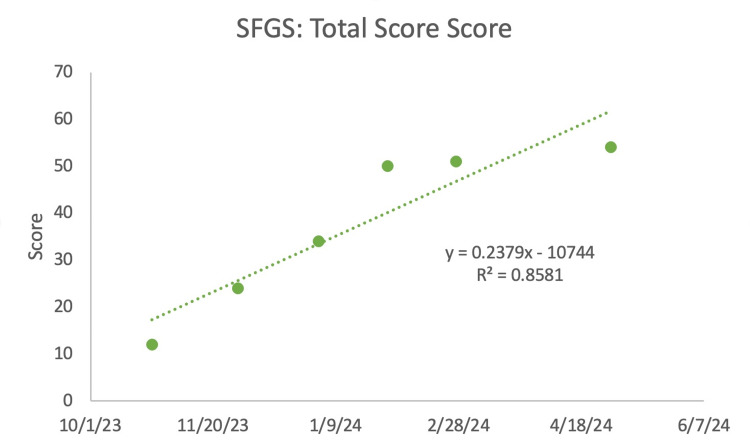
Sunnybrook facial grading system (SFGS): total score The larger the number, the better the patient's symptoms.

The overall score for the SFGS improved over the course of both OMT and PT treatments (Figure 6). There had been improvement in facial movement and less paralysis with the treatments the patient underwent.

Overall, this case study shows positive results in restoring facial paralysis in a patient with chronic BP through OMT, PT, and at-home exercises. Based on the treatments performed at her PT sessions, the techniques focused on strengthening the muscles of her face and neck whereas the OMT sessions provided relaxation and realignment to allow for decompression and relief of inflammation in the affected areas. OMT was an effective modality to include in the patient's treatment plan given its ability to restore homeostasis and optimize its self-healing mechanisms [5].

The possible limitations of this study include the overlap of treatment modalities (OMT, PT, and at-home exercises), variability in the consistency of at-home exercise performance, duration and delayed onset of treatment, spontaneous improvement, the subjectivity of the SFGS administration due to user bias, and outside factors such as stress and lack of sleep, hindering the full effects of the treatment modalities. Further studies are needed to substantiate the relationship of solely OMT and BP symptom relief.

## Conclusions

This case study illustrates that OMT, PT, and at-home exercises may improve chronic BP-related symptoms by treating cranial and muscular somatic dysfunctions of the head and neck. The treatment involved releasing tension in the fascia of the surrounding area as well as releasing muscle hypertonicity. The treatments that were most consistently used included muscle energy of the hypertonic scalenes, levator scapulae, and trapezius, intraoral direct myofascial release of the masseters, zygoma, and pterygoids, as well as OCMM. The treatment of OMT, PT, and at-home exercises resulted in improvements in both the total SFGS scale and the physical function scores of the FDI. The patient was prescribed two antivirals and a steroid prior to the OMT treatments; however, she did consistently attend PT sessions for 28 weeks.

Although it is challenging to determine whether solely OMT, PT, or at-home exercises were more beneficial for the long-term improvement of the patient, all of these methods played a significant role in the improvement of the BP symptoms. All three modalities may have provided a rehabilitative function by alleviating the BP symptoms. These modalities helped minimize residual inflammation and improved the patient's reports of stiffness and pain, bringing her closer to her original baseline. A combined approach may be necessary for treating BP symptoms, involving PT to strengthen the facial and neck muscles along with OMT to relax and release hypertonic muscles and fascial distortions. Further research is needed to substantiate evidence of OMT in the improvement of both chronic and acute BP.
